# Antigen-specific T Cell precursor frequency influences the proliferation of stem-like T Cells in tumor-draining lymph nodes and anti-tumor immune responses

**DOI:** 10.3389/fmolb.2026.1771474

**Published:** 2026-01-28

**Authors:** Liangnian Wei, Rong Chen, Kaijing Zhang, Tianyang Guo, Pengfei Li, Chunbing Zhang

**Affiliations:** 1 Department of Clinical Laboratory, Affiliated Hospital of Nanjing University of Chinese Medicine, Jiangsu Province Hospital of Chinese Medicine, Nanjing, Jiangsu, China; 2 College of First Clinical Medicine, Nanjing University of Chinese Medicine, Nanjing, Jiangsu, China; 3 State Key Laboratory of Ultrasound in Medicine and Engineering, Chongqing Medical University, Chongqing, China

**Keywords:** immunotherapy, precursor frequency, stem-like T cells, TDLN, tumor immune responses

## Abstract

**Background:**

While immune checkpoint blockade has transformed the landscape of cancer therapy, variability in patient responses constrains its therapeutic potential. Tumor-specific stem-like T (TSL) cells sustain durable immunity through self-renewal and differentiation. However, upstream regulators of TSL cells generation remain elusive, and direct evidence is still lacking regarding whether naïve tumor-specific T cell precursor frequency influences TSL cells pool establishment and immunotherapy response. This study aims to clarify the direct causal relationship between this precursor frequency and the number of TSL cells in tumor-draining lymph nodes, as well as the efficacy of anti-PD-L1 therapy.

**Methods:**

We initially constructed a novel bone marrow cell chimeric model. Specifically, recipient mice of the wild-type C57BL/6 strain were subjected to lethal irradiation, after which they received intravenous adoptive transfer of a cell suspension (5 × 10^7^ cells) comprising a blend of bone marrow cells derived from OT-I mice and wild-type mice in varying proportions. Subsequently, a subcutaneous transplanted tumor model was established by inoculating B16-OVA melanoma cells expressing the OVA antigen. Over the course of tumor progression, we evaluated tumor growth dynamics, mice survival outcomes, and T cell within the tumor-draining lymph nodes (TDLNs). Thereafter, we evaluated whether this divergence in precursor frequency of antigen-specific T cells modulates the efficacy of anti-PD-1 therapy.

**Results:**

The results of the bone marrow chimeric model demonstrated that: different precursor frequencies of antigen-specific T cell s significantly affect the abundance of stem-like T cells in the TDLNs of tumor-bearing mice. A higher precursor frequency of antigen-specific T cell could significantly suppress tumor growth and prolong the survival time of the mice. In the group with a higher precursor frequency, anti-PD-1 therapy effectively inhibited tumor growth, leading to long-term survival of the mice.

**Conclusion:**

Our study provides the first direct *in vivo* evidence that the initial precursor frequency of tumor-antigen-specific T cell influences the stem-like T cell pool in TDLNs and thereby the efficacy of anti-PD-1 immunotherapy. This finding reveals a key upstream mechanism of response heterogeneity and offers a strategic rationale for overcoming ICB resistance.

## 1 Introduction

Immune checkpoint blockade (ICB) therapy, targeting pathways such as PD-1/PD-L1, has revolutionized advanced cancer treatment by enabling durable anti-tumor immunity and long-term survival in some patients (Tumeh et al., 2014; Sharma et al., 2023). However, primary or acquired resistance remains prevalent, making response heterogeneity a major clinical challenge (Sharma et al., 2023; Vesely et al., 2022). Thus, identifying the determinants of differential efficacy is crucial for improving patient outcomes. Durable ICB response depends on stem-like T cells (TSL), a self-renewing population marked by TCF-1 and PD-1 expression in sites like tumor-draining lymph nodes (Schenkel et al., 2021; Gebhardt et al., 2023). These cells sustain anti-tumor immunity by generating effector T cells, making the TSL cells pool a key correlate of treatment success (Connolly et al., 2021; Siddiqui et al., 2019). Nevertheless, the upstream mechanisms controlling TSL cells pool establishment and size are poorly understood.

There exists a persistent yet inadequately validated hypothesis that the precursor frequency of naïve, tumor-antigen-specific T cells within the host’s lymphoid compartment serves as a fundamental bottleneck limiting the subsequent anti-tumor immune response (Rizzuto et al., 2009). Naturally, an expanded baseline pool of antigen-specific precursors should increase the probability of efficient T cell priming in tumor-draining lymph nodes, thereby laying the groundwork for a more robust TSL cells population (Connolly et al., 2021; Huang et al., 2022). This precursor frequency, shaped by the individual’s T cell repertoire, could represent a key intrinsic variable explaining inter-patient heterogeneity in ICB efficacy (Umeshappa et al., 2013; Malandro et al., 2016). However, direct *in vivo* experimental evidence causally linking the physiological precursor frequency of naïve tumor-specific T cell to the size of the TSL cells pool and, ultimately, to the therapeutic outcome of PD-1/PD-L1 blockade has been conspicuously lacking.

To address this issue, we designed a study to formally test the causal relationship between tumor-antigen-specific T cell precursor frequency and the efficacy of anti-PD-L1 immunotherapy. We employed a novel bone marrow chimeric mouse model that allows precise titration of naïve precursor frequency within a polyclonal immune system. Our findings provide direct evidence that the initial precursor frequency dictates the size of the TSL cells reservoir in TDLNs and is a decisive determinant of response to anti-PD-L1 therapy. This work elucidates a fundamental upstream mechanism of ICB response heterogeneity and highlights the therapeutic potential of strategies aimed at modulating the precursor pool to overcome resistance.

## 2 Methods

### 2.1 Cells

B16F10-OVA cells were grown in Gibco DMEM supplemented with 10% fetal bovine serum (Gibco) under 5% CO_2_ incubation conditions. *Mycoplasma* negativity of all cells was confirmed by PCR analysis before their use in experiments.

### 2.2 Mice

8-week-old female C57BL/6 mice were purchased from the Animal Experiment Center of Nanjing Medical University. CD45.1^+^ OT-1 mice were obtained from Dr. Xiaoming Wang’s laboratory. All mice were housed under specific pathogen-free conditions.

### 2.3 Tumor model

Female C57BL/6 mice (8 weeks of age) were administered 7 Gy of total-body X-ray irradiation. Within 24 h post-irradiation, the animals received an intravenous injection of 5 × 10^7^ bone marrow cells from WT C57BL/6 mice, in combination with a predetermined number of CD45.1^+^ bone marrow cells isolated from OT-1 mice. Following an 8-week interval, 5 × 10^5^ B16F10-OVA cells were injected subcutaneously into the mice. We measured the length and width of tumors, and tumor volume was computed using the formula: [width^2^ × length]/2. Mice were deemed deceased when their tumor volume reached 1800 mm^3^. For combination immunotherapy evaluation, tumor-bearing mice were intraperitoneally injected with 100 μg anti-PD1 antibody (BioXcell, clone: RMP1-14) on day 7, followed by injections every 3 days for a total of five doses.

For the splenocyte adoptive transfer model: Eight-week-old female C57BL/6 mice were subcutaneously inoculated with 5 × 10^5^ B16F10-OVA cells. Fifteen days later, the mice received 7 Gy total-body irradiation and, within 24 h, were intravenously transferred with 5 × 10^7^ splenocytes from WT C57BL/6 mice plus a specified number of CD45.1^+^ OT-1 cells. Tumor growth was recorded.

### 2.4 Flow cytometry

To evaluate the precursor frequency of antigen-specific T cell in mice, we collected 50 μL of peripheral blood from mice at 8 weeks after bone marrow transplantation. Cells were stained with anti-CD45-BV605, anti-CD45.1-BV510, anti-CD8a-AF700, and anti-TCRαV2-FITC antibodies (Biolegend) at 4 °C for 30 min, lysed with ACK buffer, and analyzed on a BD A1 flow cytometer.

For draining lymph node analysis, TDLNs were collected at day 16 post-tumor inoculation for flow cytometric analysis. Inguinal lymph nodes were harvested from tumor-bearing mice, minced, and filtered through a 70-μm strainer. After washing with PBS containing 2% FBS, cells were stained with anti-CD45.1-BV510, anti-CD8-AF700, and anti-CD366-BV605 (Biolegend) at 4 °C for 30 min, fixed and permeabilized using the Foxp3/Transcription Factor Staining Buffer Set (Invitrogen), and then incubated with anti-TCF7/TCF1-APC (clone C63D9, Cell Signaling Technology) at 4 °C for 40 min. Samples were analyzed on a BD A1 flow cytometer.

### 2.5 Statistical analysis

Flow cytometry data were analyzed via FlowJo v10.8.1. GraphPad Prism 10 served for statistical analysis and graph generation, with group differences evaluated by one-way ANOVA and survival outcomes compared using the log-rank test.

## 3 Result

### 3.1 The precursor frequency of antigen-specific T cell affects tumor growth and the TSL cells frequency of TDLNs in bone marrow chimeric mice

**FIGURE 1:**
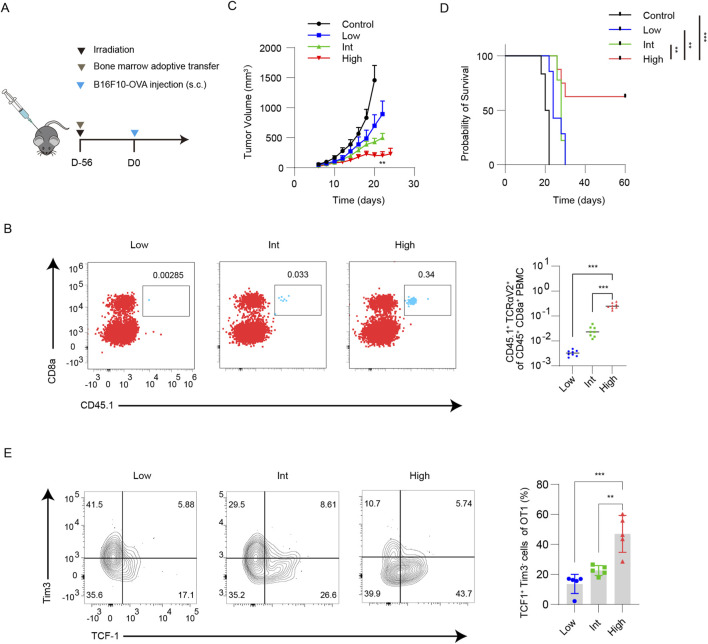
The precursor frequency of antigen-specific T cell affects tumor growth and the TSL cells frequency of TDLNs in bone marrow chimeric mice. **(A)** Lethally irradiated C57BL/6 mice were reconstituted with WT bone marrow supplemented with 1 × 10^3^, 1 × 10^4^, or 1 × 10^5^ CD45.1^+^ OT-I bone marrow cells (Low, Int, High groups, respectively). **(B)** Flow cytometric analysis of CD45.1^+^CD8^+^TCRαV2^+^ cells verified graded antigen-specific precursor frequencies, with quantitative results presented. **(C)** Subcutaneous B16F10-OVA tumor growth was significantly suppressed in the High group compared with the Int and Low groups (n = 8). **(D)** The long-term survival rate was increased in the High group (5/8). **(E)** Flow cytometric analysis of TDLNs showed the proportion of TCF-1^+^Tim-3^-^ OT-I cells, with quantitative results presented (n = 5). Data are presented as mean ± SD. Statistical significance was determined by one-way ANOVA with Tukey’s multiple comparisons test **(B,C,E)** and log-rank test **(D)**. *P < 0.05, **P < 0.01, ***P < 0.001.

To better simulate the impact of different antigen-specific T cell precursor frequencies on tumor growth and the stem-like T cell pool, we established mouse models with varying precursor frequencies of antigen-specific T cell using a bone marrow adoptive transfer strategy. Lethally irradiated C57BL/6 mice were reconstituted with 5 × 10^7^ WT mouse bone marrow cells supplemented with either 1 × 10^3^ (Low group), 1 × 10^4^ (Int group), or 1 × 10^5^ (High group) CD45.1^+^ OT-1 mice bone marrow cells (Figure 1A). Eight weeks later, successful reconstitution was confirmed by flow cytometric analysis of OT-I cell markers (CD45.1^+^CD8^+^TCRαV2^+^). The Low, Int, and High groups exhibited approximately 3.3 × 10^−3^%, 2.6 × 10^−2^%, and 2.6 × 10^−1^% antigen-specific T cell precursors, respectively (Figure 1B). A frequency of ∼0.003% more closely approximates the physiological precursor frequency for many tumor antigens. Additionally, the gradient allowed us to test whether subtle differences in precursor abundance could meaningfully impact TSL cells pool formation and therapeutic outcome. Subsequently, a subcutaneous tumor model was established using B16F10-OVA cells. Tumor growth analysis revealed that increasing precursor frequency significantly suppressed tumor progression (Figure 1C). In the High group, over half of the mice (5/8) achieved long-term survival (Figure 1D). In this study, we identified stem-like T (TSL) cells based on the co-expression of TCF-1 and the absence of Tim-3, a phenotype widely associated with self-renewal capacity, multipotency, and the ability to sustain long-term anti-tumor responses in multiple tumor models, including melanoma (Burger et al., 2021; Li et al., 2025; Onyshchenko et al., 2024). Flow cytometric analysis of tumor-draining lymph nodes showed that the proportion of TCF-1^+^Tim-3^-^ antigen-specific T cells was significantly higher in the High group compared to the Int and Low groups (Figure 1E). These data indicate that a higher precursor frequency of antigen-specific T cell significantly inhibits tumor growth and enriches the proportion of stem-like T cells within tumor-draining lymph nodes.

### 3.2 Antigen-specific T-cell precursor frequency regulates tumor growth and the frequency of TSL cells in a splenocyte adoptive transfer model

**FIGURE 2:**
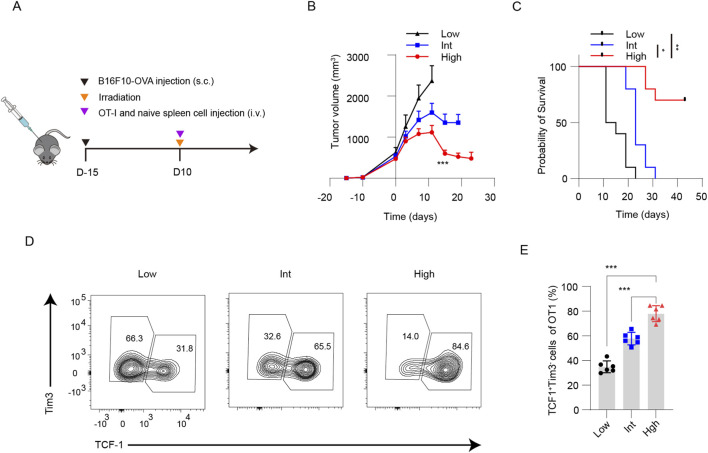
Antigen-specific T-cell precursor frequency regulates tumor growth and the frequency of TSL cells in a splenocyte adoptive transfer model. **(A)** C57BL/6 mice bearing 15-day established subcutaneous B16F10-OVA tumors were lethally irradiated, followed by adoptive transfer of 5 × 10^7^ WT splenocytes plus 1 × 10^3^, 1 × 10^5^, or 1 × 10^6^ CD45.1^+^ OT-1 splenocytes (Low, Int, High groups, respectively). **(B)** Tumor growth was significantly inhibited with increasing antigen-specific T-cell precursor frequency (n = 10). **(C)** The long-term survival rate was elevated in the High group (7/10). **(D)** Representative flow cytometric plots of TCF-1^+^Tim-3^-^ OT-I cells in TDLNs. **(E)** Quantitative analysis showing a significantly higher proportion of TCF-1^+^Tim-3^-^ TSL cells in the High group compared with the Int and Low groups (n = 6). Data are presented as mean ± SD. Statistical significance was determined by one-way ANOVA with Tukey’s multiple comparisons test **(B,E)** and log-rank test **(C)**. *P < 0.05, **P < 0.01, ***P < 0.001.

To further validate the impact of antigen-specific T-cell precursor frequency on tumor growth and the stem-like T-cell pool, we established a splenocyte adoptive transfer model (Rizzuto et al., 2009). C57BL/6 mice bearing established (15-day) subcutaneous B16F10-OVA tumors received lethal irradiation, followed by adoptive transfer of 5 × 10^7^ WT mouse splenocytes together with 1 × 10^3^ (Low group), 1 × 10^5^ (Int group), or 1 × 10^6^ (High group) CD45.1^+^ OT-1 splenocytes (Figure 2A). Tumor growth analysis demonstrated that increasing precursor frequency significantly inhibited tumor progression (Figure 2B). In the High group, over half of the mice (7/10) achieved long-term survival (Figure 2C). Flow cytometric analysis of tumor-draining lymph nodes revealed that the proportion of TCF-1^+^Tim-3^-^ antigen-specific T cells was significantly higher in the High group compared to the Int and Low groups (Figures 2D,E). These data further indicate that a higher precursor frequency of antigen-specific T-cells significantly suppresses tumor growth and enriches the pool of stem-like T cells in tumor-draining lymph nodes.

### 3.3 The increased precursor frequency of antigen-specific T-cell enhances responsiveness to anti-PD-1 immunotherapy

**FIGURE 3:**
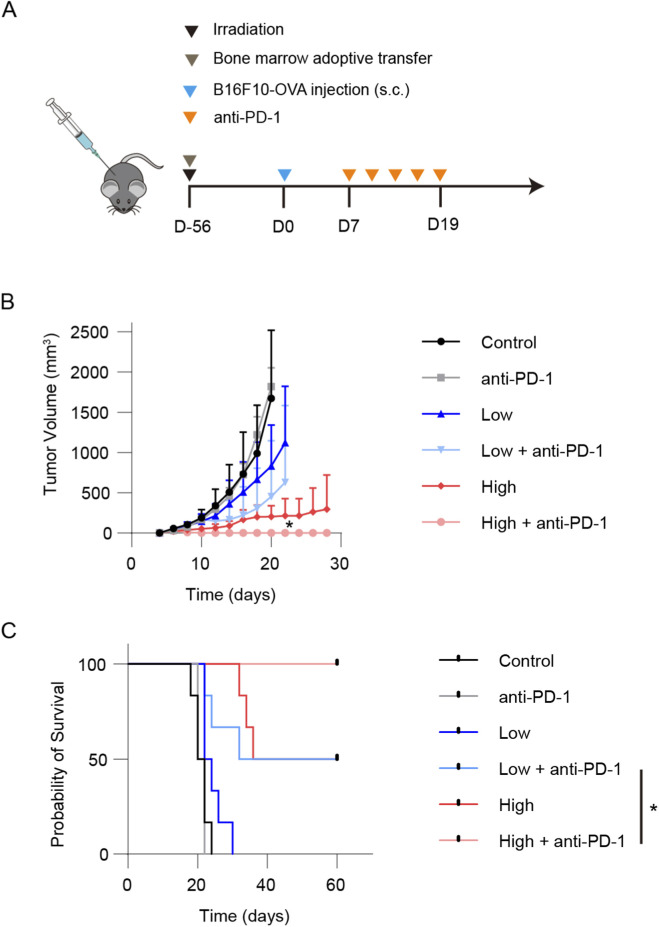
Increased antigen-specific T-cell precursor frequency enhances responsiveness to anti-PD-1 immunotherapy. **(A)** Schematic of the experimental design: following bone marrow adoptive transfer, mice were administered five doses of anti-PD-1 therapy. **(B)** Tumor growth curves showing that the high antigen-specific CD8^+^ T-cell precursor frequency group achieved a complete response to anti-PD-1 treatment (n = 6). **(C)** Survival analysis demonstrates that all mice in the high precursor frequency group attained long-term survival (6/6). Data are presented as mean ± SD. Statistical significance was determined by one-way ANOVA with Tukey’s multiple comparisons test **(B)** and log-rank test **(C)**. *P < 0.05, **P < 0.01, ***P < 0.001.

Finally, to assess whether the enrichment of stem-like T cells in tumor-draining lymph nodes resulting from an increased antigen-specific T-cell precursor frequency alters the efficacy of immune checkpoint blockade, we investigated the impact of different precursor frequencies on immunotherapy. Following bone marrow adoptive transfer, mice received five doses of anti-PD-1 treatment (Figure 3A). Tumor growth analysis showed that the group with a high precursor frequency of antigen-specific CD8^+^ T-cells achieved a complete response to anti-PD-1 therapy (Figure 3B), with all mice (6/6) attaining long-term survival (Figure 3C). These data demonstrate that an increased frequency of antigen-specific T-cell precursors significantly enhances responsiveness to immunotherapy.

## 4 Discussion

We demonstrate in our research that an elevated initial precursor frequency of tumor-antigen-specific T cell substantially affects the TSL cells pool within TDLNs. Ultimately, this alteration changes the effectiveness of anti-PD-1 immunotherapeutic intervention. This central conclusion, validated across both bone marrow chimeric and splenocyte adoptive transfer models, provides a crucial upstream mechanistic explanation for the heterogeneity observed in immunotherapy responses. We obtained direct evidence from *in vivo* models: the baseline abundance of naïve antigen-specific T cell precursors is a key regulator of immune response capacity. Individuals exhibit naturally occurring variation in their T cell repertoires, and this divergence may result in significant discrepancies in precursor frequency for particular tumor neoantigens. Our findings indicate that this variation (simulated across a frequency gradient of ∼0.003%–∼0.26%) modulates the size of the TSL cells pool in TDLNs and the capacity for tumor control. This offers a novel explanation for the clinical heterogeneity in ICB efficacy: some patients may lack a sufficient number of initial T cell “seeds” capable of being effectively primed and differentiated into functional TSL cells (Connolly et al., 2021; Huang et al., 2022). Consequently, even upon relieving immune suppression with ICB, an effective anti-tumor response cannot be mounted. Therefore, assessing antigen-specific precursor frequency holds promise as a novel biological biomarker for predicting ICB response (Zehn et al., 2022).

Study specifically identifies the TDLN as the critical site for TSL cells enrichment (Prokhnevska et al., 2023). This structure serves as the primary venue where naïve T cells encounter tumor antigen, undergo priming, and initiate early differentiation (Prokhnevska et al., 2023; Delclaux et al., 2024). A higher precursor frequency increases the probability of productive interactions between T cells and antigen-presenting cells, potentially leading to superior priming signals (e.g., co-stimulation, cytokines) (Burger et al., 2021). This favors the programming and maintenance of the TCF-1-driven stem-like phenotype. Conversely, under low-frequency conditions, the formation of effective immune synapses is challenging (Brewitz et al., 2017; Dahling et al., 2022). This may prevent T cells from receiving adequate signals to enter the TSL cells differentiation pathway or result in the generation of only a small number of low-quality TSL cells. Our data support the concept of the TDLN as a “processing factory,” where the output (the TSL cells pool) in both scale and quality is highly dependent on the input of raw material (precursors).

The rigor of this study is reflected in the employment of two independent models. The bone marrow chimeric model simulates a long-term, stable precursor frequency set at the hematopoietic developmental level, reflecting the host’s “baseline state.” In contrast, the splenocyte adoptive transfer model simulates the scenario of “supplementing” precursors after tumor establishment via adoptive cell therapy, offering greater relevance to immediate therapeutic intervention.

Our study also has certain limitations that warrant attention in future work. (1) The use of a single, strong model antigen (OVA) oversimplifies the complexity of tumor antigens. Human tumors possess a polyclonal neoantigen repertoire with varying immunogenicity and precursor frequencies (Obar et al., 2008; McGranahan et al., 2016). The “low” frequency group used in the experiments may still be numerically higher than the true precursor frequency for many human tumor neoantigens, which are likely even rarer (Obar et al., 2008). The lymphodepleted environment induced by irradiation can trigger homeostatic proliferation, which may artificially influence the biology of the transferred T cells (Gattinoni et al., 2005; Guilbaud et al., 2023). The conclusions require validation in a broader range of tumor models, particularly “hot” tumors or spontaneous tumor models. The study primarily focuses on CD8^+^ T cells. Specific regulatory effects are associated with other immune components, like CD4^+^ T cells and regulatory T cells (Tregs). These functions remain to be further clarified. (Chen et al., 2024; Hu et al., 2022; Bhandarkar et al., 2025). From a translational perspective, our findings suggest that strategies aimed at expanding the pool of tumor-specific naïve T cell precursors—such as through neoantigen vaccination, cytokine modulation, or *ex vivo* enrichment for adoptive cell therapy—could serve as rational combinatorial approaches to overcome ICB resistance. Assessing tumor-specific T cell precursor frequency in patients may offer predictive value for ICB response. Techniques such as peptide-MHC multimer staining, TCR sequencing of naïve or memory subsets, and functional assays like ELISpot could be deployed to quantify pre-existing tumor-reactive clones. Our data support the rationale for developing these precursor frequency-based biomarkers to guide personalized immunotherapy.

## 5 Conclusion

In summary, this study identifies the initial tumor-antigen-specific T cell precursor frequency as a key factor influencing the establishment of the TSL cells pool and the efficacy of PD-1 blockade. It traces the differences in immunotherapy response back to the most fundamental “inventory” level of the immune system. This not only deepens our understanding of the mechanisms underlying response heterogeneity but, more importantly, points clinical practice toward a new paradigm: shifting from passively “releasing inhibition” to actively “laying the foundation and expanding” the immune response. Future research should focus on translating this principle into actionable clinical tools and strategies, ultimately aiming for the precision and broad applicability of immunotherapy.

## Data Availability

The original contributions presented in the study are included in the article/supplementary material, further inquiries can be directed to the corresponding authors.
